# Why 2 Studies That Used the Same Probiotic May Have Come Up With Different Outcomes

**DOI:** 10.1093/cid/ciab618

**Published:** 2021-09-16

**Authors:** Lynne V McFarland, Ravina Kullar, Pierre-Jean Maziade, Ellie J C Goldstein

**Affiliations:** 1 Department of Medicinal Chemistry, University of Washington, Seattle, WashingtonUSA; 2 Expert Stewardship Inc, Newport Beach, California, USA; 3 Department of Microbial and Infectious Disease, Centre Integre de Sante et de Services Sociaux de Lanaudiere, Terrebonne, Canada; 4 R.M. Alden Research Laboratory, Los Angeles, California, USA

To the Editor—Two recent studies tested the same 3-strain Lactobacillaceae probiotic mixture for the prevention of *Clostridium difficile* infection (CDI), yet reported conflicting results [1, 2]. In studies that test probiotics for CDI prevention, different efficacies are often reported, but typically it is due to not accounting for the strain-specific efficacy of probiotics [3]. In this case, 2 quasiexperimental studies (QES) tested the same type of probiotic using an electronic decision support tool that triggered a flag for probiotic use for eligible inpatients receiving antibiotics and then compared CDI rates during the intervention to a control time period prior to the intervention. A review of 28 QES found that limitations included low implementation of the probiotic and not accounting for changes in infection control measures or antibiotic use during the 2 time periods [4]. With these limitations in mind, we examined these 2 studies to attempt to determine why the same probiotic was effective in the Maziade et al study [1] but not in the Heil et al study [2].

Both QES designs used the same probiotic, the same dose (10^11^/day), and the same duration (during antibiotics use plus 5 days afterward), and the electronic orders were triggered within 12–24 hours of the first antibiotic dose. Both studies compared hospital-onset CDI level data and patient-level data and also adjusted risk estimates for CDI risk factors.

Differences in the 2 studies (Table 1) show different trends in CDI rates during the control period, increasing in one study [1] and decreasing in the other [2]. During the intervention period, CDI rates significantly decreased in one study [1] but increased in the other study [2]. Similar results were seen in the patient-level data, and adjustment for CDI risk factors resulted in nonsignificant differences the in Heil et al study [2] but significant efficacy remained in the Maziade et al study [1].

**Table 1.** Comparison of Study Design Factors and Results of 2 Quasiexperimental Studies That Implemented a 3-Strain Probiotic for the Prevention of *Clostridiodies difficile* Infections

Factor/Outcome	Heil et al	Maziade et al
CDI rate during		
Control period	Decreased	Increased
Probiotic intervention period	Increased	Decreased
Hospital-wide CDI rate (per patient-days)		
Control period	1/10 000^a^	8.6/10 000
Probiotic intervention period	2.5/10 000^a^	5.2/10 000^b^
Patient-level CDI rate		
Control period	132/17 536 (0.75%)^c^	84/5666 (1.5%)^d^
Probiotic intervention period	153/15 023 (1.1%)^c,^^b^	73/8266 (0.9%)^d^^,^^b^
Adjusted CDI risk estimate (95% confidence interval)	1.46 (0.87–2.45)	0.42 (0.28–0.63)^b^
Intervention implementation		
Electronic order triggered^c^	5203/15 023 (35%)	6079/6079 (100%)
Received probiotic^c^	2489/15 023 (17%)	4543/6079 (75%)

The 3-strain probiotic was (*Lactobacillus acidophilus* CL1285, *Lacticaseibacillus* [*Lactobacillus*] *casei* LBC80R, and *Lacticaseibacillus* [*Lactobacillus*] *rhamnosus* CLR2.

Abbreviation: CDI, *Clostridium difficile* infection.

^a^Estimated from Figure data in Heil et al., no raw hospital-level data reported.

^b^ *P* < .05 compared with control period.

^c^Among eligible inpatients.

^d^ Among number of patient visits.

Other factors that may influence CDI rates during the 2 study time periods were also compared, but the rates of antibiotic use, types of antibiotic used, age of inpatients, changes in infection control practices, and similar factors did not explain why the CDI increased in one study and decreased in the other. Part of the intervention period for the most recent study [2] did occur during the coronavirus disease 2019 pandemic, when increased antibiotic use was being observed [5]. However, in 3 of the 4 hospitals in the Heil et al study, the rates of CDI increased in January 2020, a few months before the pandemic.

The most significant difference between these 2 studies was the degree of successful implementation of the probiotic intervention (Table 1). The electronic tool was triggered for 100% of eligible inpatients in the Maziade et al study [1] but was triggered for only 35% in the Heil et al study [2]. Additionally, while 75% of eligible patients actually received the probiotic in one study [1], only 17% received the probiotic in the other [2]. For a probiotic intervention to have a significant impact on hospital-wide CDI rates, this shows the importance of the degree of penetration that an intervention needs to achieve. This might explain the markedly different results.

## Note

***Potential conflicts of interest*.** L. V. M., E. J. C. G., R. K., and P.-J. M. serve on the Bio-K Plus International, Inc, Scientific Advisory Board. L. V. M. reports consulting fees for writing articles, as requested, and honoraria for annual scientific advisory meeting attendance from Bio-K Plus International, Inc, outside the submitted work. E. J. C. G. reports grants/payment for serving on an advisory board for Bio-K Plus International, Inc, outside the submitted work. R. K. reports receiving honoraria for serving on an advisory board for Bio-K Plus International, Inc, outside the submitted work. P.-J. M. reports serving on a scientific advisory board for Bio-K Plus International, Inc, outside the submitted work. All authors have submitted the ICMJE Form for Disclosure of Potential Conflicts of Interest. Conflicts that the editors consider relevant to the content of the manuscript have been disclosed.
